# Comprehensive Analysis of the Oncogenic, Genomic Alteration, and Immunological Landscape of Cation-Chloride Cotransporters in Pan-Cancer

**DOI:** 10.3389/fonc.2022.819688

**Published:** 2022-03-17

**Authors:** Jie Wang, Wangrui Liu, Wenhao Xu, Baofeng Yang, Mingzhu Cui, Zhen Li, Hailiang Zhang, Chuntao Jin, Huanzhou Xue, Jiaqiang Zhang

**Affiliations:** ^1^ Department of Anesthesiology and Perioperative Medicine, People’s Hospital of Zhengzhou University, Henan Provincial People’s Hospital, Zhengzhou, China; ^2^ Department of Neurosurgery, Affiliated Hospital of Youjiang Medical University for Nationalities, Baise, China; ^3^ Department of Urology, Fudan University Shanghai Cancer Center, Shanghai, China; ^4^ Department of Anesthesiology and Perioperative Medicine, Affiliate Cancer Hospital of Zhengzhou University, Zhengzhou, China; ^5^ Department of Pathology, Henan Provincial People's Hospital, People's Hospital of Zhengzhou University, Zhengzhou, China; ^6^ Department of Hepatobiliary Surgery, People’s Hospital of Zhengzhou University, Henan Provincial People’s Hospital, Zhengzhou, China

**Keywords:** cation-chloride cotransporters (CCCs), KCC2, NKCC1, pan-cancer analysis, prognosis, tumor microenvironment (TME), DNA variation

## Abstract

**Background:**

Assessing the phenotypic diversity underlying tumor progression requires the identification of variations in the respective molecular interaction in the tumor microenvironment (TME). Despite emerging studies focusing on the association between cation-chloride cotransporters (CCCs) and carcinogenesis, direct evidence that CCCs (KCC2 and NKCC1) mediate tumor progression in pan-cancer remains unclear.

**Methods:**

We conducted a comprehensive assessment of the expression, DNA variation profiles, and prognostic and immunologic implications of CCCs based on a large-scale pan-cancer population, including 10,967 cancer patients from the Cancer Genome Atlas, 9,162 cancer patients from Genomics Expression Omnibus, 48,834 cancer patients from 188 independent studies, and 356 cancer patients from three real-world cohorts.

**Results:**

In this study, we first found that CCCs were highly expressed in most tumors, and prominently associated with prognosis. Kaplan–Meier analysis and Cox regression analysis revealed that KCC2 and NKCC1 significantly predicted survival for patients with pan-cancer, suggesting that CCCs have inconsistent tumorigenesis regulatory mechanisms in cancers. Next, we examined the DNA variation landscape of KCC2 and NKCC1 and their prognostic implications in pan-cancer. The results demonstrated that UCEC patients with somatic copy number variation (CNV) of NKCC1 received significantly better outcomes (*p <* 0.05). Besides emphasizing the clinical implications of CNV of CCCs for cancer patients, we found that NKCC1^MUT^ could prominently prolong progression-free survival (*p* = 2.59e-04), disease-specific survival (*p* = 0.019), and overall survival (*p* = 0.034) compared with NKCC1^WT^ cancer patients possibly *via* regulation of cell proliferation and oncogenic stress pathways. Additionally, KCC2 positively correlated with the levels of tumor-infiltrating macrophages and CD4^+^ T cells, but NKCC1 showed a significantly widely negative association with tumor-infiltrated lymphocytes, suggesting an immune-excluded TME in cancers. Similarly, expression of KCC2, rather than NKCC1, was positively correlated with the immune checkpoint molecules, indicating its role as an immune regulator in a wide variety of cancers. Finally, to verify our hypothesis and altered expression of CCCs, we performed IHC analysis and revealed the staining distribution in tumor and adjacent normal tissues of glioma, clear cell renal cell carcinoma, papillary cell renal cell carcinoma, and hepatocellular and breast cancer from three real-world cohorts, and validated prominently prognostic implications of CCCs in patients with clear cell renal cell carcinoma.

**Conclusion:**

This study first comprehensively investigated the molecular and clinical role of CCCs, and illustrated the significant association among KCC2/NKCC1 expression, DNA variation profiles prognosis, and TME of pan-cancer. The pan-cancer findings provided an in-depth understanding of potential oncogenic and immunologic of differential expression and DNA alteration of KCC2/NKCC1 cancers.

## Introduction

Cation-chloride cotransporters (CCCs) regulate the osmotic pressure and water balance inside and outside the cell membrane (1, 2), and promote the neutralization and transport of electrons inside and outside the cell membrane, such as K^+^, Na^+^, and C1^-^ (3, 4). CCCs are composed of Na^+^/K^+^ coupled transporters, which include NCC, NKCC1, and NKCC2; the K+ coupling transporter group is composed of KCC family genes (KCC1–4) (5, 6). Studies have shown that CCCs are involved in a variety of physiological processes in the human body, including epithelial ion absorption, cell volume regulation, and chlorine homeostasis, and could promote development and worsening invasion of cancer cells (7, 8).

With the deepening of research, tumor cell-unlimited proliferation and strong invasive and metastasis ability are the main causes of high malignancy degree and aggressive prognosis (9, 10). These processes, including regulation of tumor progression, proliferation, invasion, and metastasis, involve cell surface molecules, epithelial cytoskeleton and cell membranes (11–13). Increasing studies have found that ion channels and transporters play a key role in the invasion of tumor cells. For example, permeation-related ions allow tumor cells to invade by adjusting the shape and volume of cells. Besides, to adequately assess a biomarker’s ability to predict future risk of neoplastic progression, researchers have explored the prognostic and predictive immunological implications of cancers, such as glioma (14), liver carcinoma (15), and renal cell carcinoma (16). However, specifically, identifying highly sensitive and specific tumor markers is a challenge that needs to be solved for the early diagnosis of pan-cancer. Therefore, the prognostic and immunological roles of ion channel-related signatures in cancers were required for understanding cation-chloride transportation, predicting prognosis, and optimizing treatment selection for cancer patients.

With the rapid development of modern technology, bioinformatics and computational biology have recently been utilized for the profiling of tumor tissues to assist the investigators in developing novel insights into anti-tumor studies (17–19). Many studies have also found that ion channels are associated with malignant biological behaviors in cancer cells, including progressive proliferation and invasion, cell cycle changes, and angiogenesis disorders (20, 21). In this study, we hypothesized that KCC2 (SLC12A5) and NKCC1 (SLC12A2), the major cation-chloride transporters, could predict the occurrence and development of cancers by regulating ion channels in pan-cancer, which may provide promising potential targets for clinical management strategies.

## Methods

### Cancer Sample Collection and Data Pre-Processing

The RNA-seq, copy number variation, mutation, clinical, and survival data of 10,967 pan-cancer patients were obtained from The Cancer Genome Atlas (TCGA, https://portal.gdc.cancer.gov) database (22). In addition, the data of normal tissue samples were included from GTEx V8 version (https://gtexportal.org/home/datasets), and the clinical pathological information of the participants was fully described in the official GTEx annotation. Besides, 48,834 cancer samples with available DNA variation profiles from 188 independent cohorts were enrolled using cBioPortal for Cancer Genomics (http://www.cbioportal.org/) in this study to investigate the genomics alteration role of KCC2 (SLC12A5) and NKCC1 (SLC12A2) in cancers. Genomics Expression Omnibus (GEO) database provided 875 gastric cancer patients, 1,925 lung cancer patients, 1,435 ovarian cancer patients, and 4,929 breast cancer patients with available RNA sequencing data, and follow-up survival information was enrolled in this study. Additionally, three real-world cohorts, People’s Hospital of Zhengzhou University (PHZU, Henan), Affiliated Hospital of Youjiang Medical University for Nationalities (AHYMUN, Guangxi), and Fudan University Shanghai Cancer Center (FUSCC, Shanghai), provided 20 samples of LIHC (liver hepatocellular carcinoma), 33 samples of BRCA (breast invasive carcinoma), 40 samples of LGG (brain lower grade glioma), 40 samples of GBM (glioblastoma multiforme), 70 KIRC (kidney renal clear cell carcinoma) samples, 50 KIRP (kidney renal papillary cell carcinoma) samples, and 50 normal kidney tissue samples for further experimental validation. All of the study designs and test procedures were performed in accordance with the Helsinki Declaration II.

### Immunohistochemistry Staining Analysis

Immunohistochemical analysis (IHC) was implemented with an anti-KCC2 (ab259969, Abcam, USA) and anti-NKCC1 (ab140986, Abcam, USA) at 1/500 dilution. IHC staining was performed according to the manufacturer’s instructions as previously described (23). On the basis of the integration of IHC staining degree of intensity and density, two experienced and independent pathologists/clinicians evaluated the overall IHC score (from 0 to 12), defining a negative staining of 0 to 3 and a positive staining of 4 to 12 for each tissue.

### Differential Expression and Survival Analysis

To illustrate the statistical significance of differential KCC2/NKCC1 expression between tumor and normal tissues, unpaired Student’s *t*-test was performed to compare the differences among cancers. The Kaplan–Meier method in 95% confidence interval (95% CI) and log-rank test was implemented to evaluate the significance of disease-specific survival (DSS) and overall survival (OS) benefits in a separate KCC2/NKCC1 expression group and all subgroups classified by tumor microenvironment infiltration characteristics using Kaplan–Meier Plotter (https://kmplot.com/analysis/). Univariate and multivariate Cox regression analysis was performed to identify the proper terms to build the Nomogram. The forest was used to show the *p*-value, HR, and 95% CI of each variable through “forestplot” R package (24). A Nomogram was developed based on the results of multivariate Cox proportional hazards analysis to predict the X-year overall recurrence to calculate the risk of recurrence for individual patients. Moreover, a Sankey diagram was utilized to explore the relationship between pathological factors and survival.

### Abundance and Frequency of KCC2/NKCC1 Mutation of HCC

To further investigate the role of KCC2 (SLC12A5) and NKCC1 (SLC12A2) in pan-cancers, we analyzed mutation abundance and frequency of KCC2/NKCC1 in pan-cancer using cBioportal for Cancer Genomics (http://www.cbioportal.org/) (25). A total of 48,834 cancer samples with available DNA variation, including mutations, copy number variation (CNV), somatic number variation (SNV), and profiles from 188 independent curated of non-redundant cohorts were enrolled. The frequency of typical gene mutations in HCC according to differential KCC2/NKCC1 expression was also analyzed after curated. Significantly elevated genes between KCC2/NKCC1 altered and unaltered groups were screened and identified using the Limma R package (26).

### Immune Infiltration Analysis of Tumor Microenvironment

Tumor Immune Estimation Resource 2.0 (TIMER 2.0, http://timer.cistrome.org/) was utilized to analyze the comprehensive correlation between abundance of tumor-infiltrating immune cells and KCC2 (SLC12A5) and NKCC1 (SLC12A2) expression using Pearson’s test (27).

### Establishment of a Protein–Protein Interaction Network and Functional Annotations of KCC2/NKCC1-Related PPI networks

Spearman’s correlation analysis was performed to describe the correlation between quantitative variables without a normal distribution. The gene ontology (GO) database was used for functional enrichment analyses of biological processes (BP), molecular functions (MF), and cellular components (CC).

### Statistical Analysis

All statistical analyses were performed using SPSS software (version 23.0, Inc, Chicago, IL) (28), GraphPad Prism 8.0, R software (version 3.4.3), or online webtools (29). All hypothetical analyses were two-sided, and *p* < 0.05 was considered as statistically significant.

## Results

### Differential Expression of KCC2 and NKCC1 in Pan-Cancer Based on TCGA and GTEx Database

**Figure 1:**
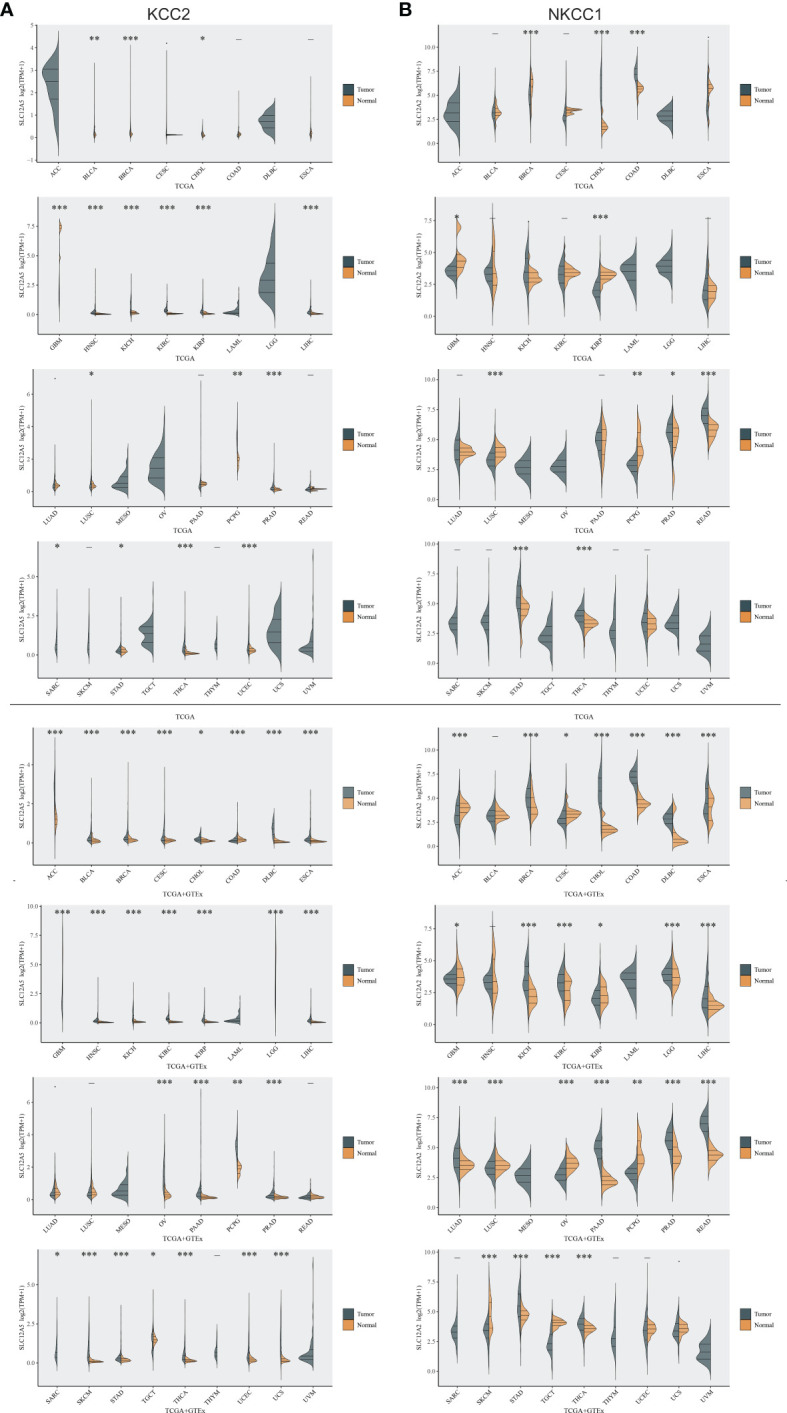
Differential expression of KCC2 and NKCC1 in pan-cancer based on a large-sample database. **(A)** The differential expression of KCC2 in pan-cancer tissues from TCGA database and normal samples from TCGA and GTEx Portal databases. **(B)** The differential expression of NKCC1in pan-cancer tissues from TCGA database and normal samples from TCGA and GTEx Portal databases. *p < 0.05, **p < 0.01, ***p < 0.001.

To investigate differential cation-chloride transportation patterns in tumors, we analyzed the differential expression of KCC2 (SLC12A5) (**Figure 1A**) and NKCC1 (SLC12A2) (**Figure 1B**) in pan-cancer tissues from TCGA database and normal samples from TCGA and GTEx Portal databases. We found significantly differential KCC2 expression in pan-cancers, such as breast invasive carcinoma, liver hepatocellular carcinoma, glioma, prostate adenocarcinoma, and uterine carcinosarcoma. Similarly, we analyzed the expression distribution of NKCC1 in the tumor and normal samples in the above-mentioned database, and found that the expression of NKCC1 also significantly differ in pan-cancer, such as cholangiocarcinoma, kidney renal papillary cell carcinoma, lung squamous cell carcinoma, kidney chromophobe carcinoma, and stomach adenocarcinoma. Overall, the transcriptional expression pattern of cation-chloride cotransporters was significant in tumor tissues, suggesting the potential involvement of ion channels in the occurrence and progression of cancers.

### KCC2 and NKCC1 Predict the Prognosis of Large-Scale Pan-Cancer

**Figure 2:**
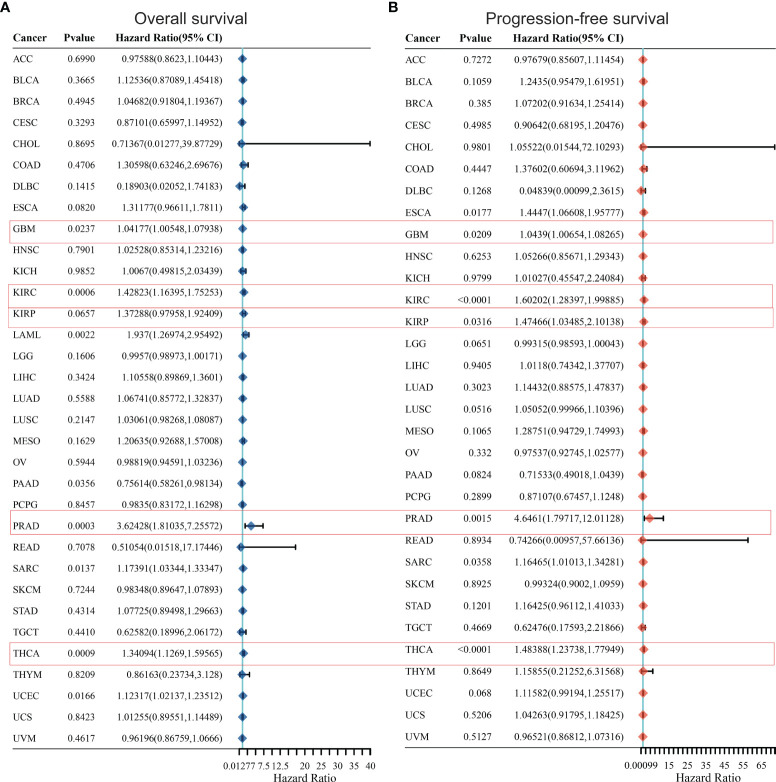
Prognostic prediction of KCC2 for overall survival **(A)** and progression-free survival **(B)** in pan-cancer. GBM (OS, *p* = 0.0237; PFS, *p* = 0.0209), KIRC (OS, *p* = 0.0006; PFS, *p* < 0.0001), PRAD (OS, *p* = 0.0003; PFS, *p* = 0.0015), and THCA (OS, *p* = 0.0009; PFS, *p* < 0.0001). GBM, Glioblastoma multiforme; KIRC, Kidney renal clear cell carcinoma; PRAD, Prostate adenocarcinoma; THCA, Thyroid carcinoma.

**Figure 3:**
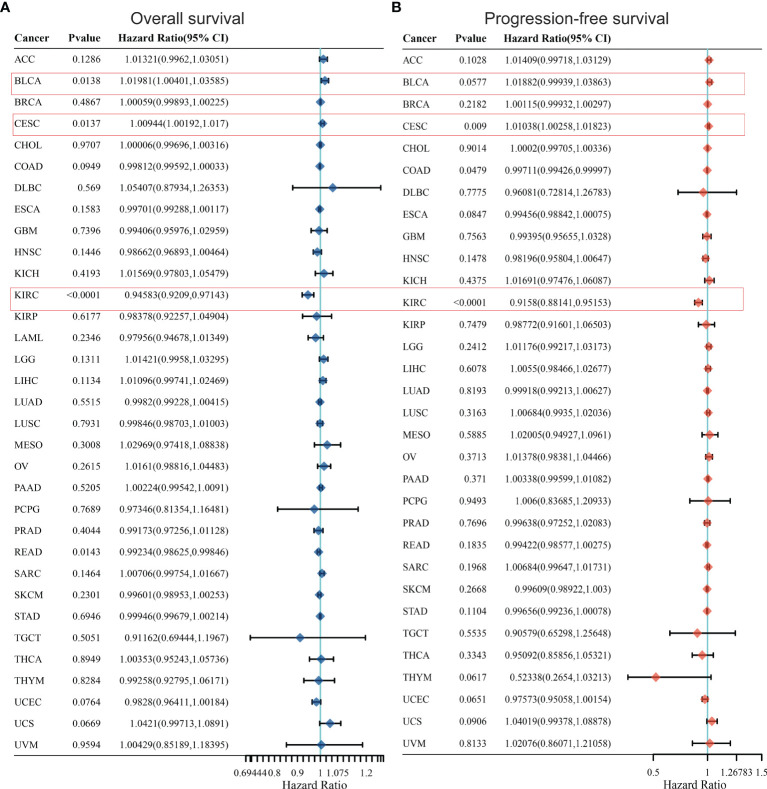
Prognostic prediction of NKCC1 for overall survival **(A)** and progression-free survival **(B)** in pan-cancer. BLCA (OS, *p* = 0.0138; PFS, *p* = 0.0577), CESC (OS, *p* = 0.0137; PFS, *p* = 0.009), and KIRC (OS, *p* < 0.0001; PFS, *p* < 0.0001). BLCA, Bladder Urothelial Carcinoma; CESC, Cervical squamous cell carcinoma and endocervical adenocarcinoma; KIRC, Kidney renal clear cell carcinoma.

In order to further optimize the prediction model and improve the clinical transformation efficiency, we performed a stepwise analysis of the impact of KCC2 (**Figures 2A, B**) and NKCC1 (**Figures 3A, B**) in pan-cancer progression-free survival (PFS) and overall survival (OS). The forest plot indicated that KCC2 expression markedly predicted prognosis in GBM (OS, *p* = 0.0237; PFS, *p* = 0.0209), KIRC (OS, *p* = 0.0006; PFS, *p* < 0.0001), PRAD (OS, *p* = 0.0003; PFS, *p* = 0.0015), and THCA (OS, *p* = 0.0009; PFS, *p* < 0.0001). NKCC1 expression was found as a significant prognostic biomarker in BLCA (OS, *p* = 0.0138; PFS, *p* = 0.0577), CESC (OS, *p* = 0.0137; PFS, *p* = 0.009), and KIRC (OS, *p* < 0.0001; PFS, *p* < 0.0001). Taken together, cation-chloride cotransporters KCC2 (SLC12A5) and NKCC1 (SLC12A2) have a certain impact on the survival of patients in pan-cancer, especially in KIRC, GBM, PRAD, THCA, BLCA, and CESC.

### Kaplan–Meier Analysis of KCC2 and NKCC1 in Pan-Cancer Samples Based on TCGA and GEO Databases

**Figure 4:**
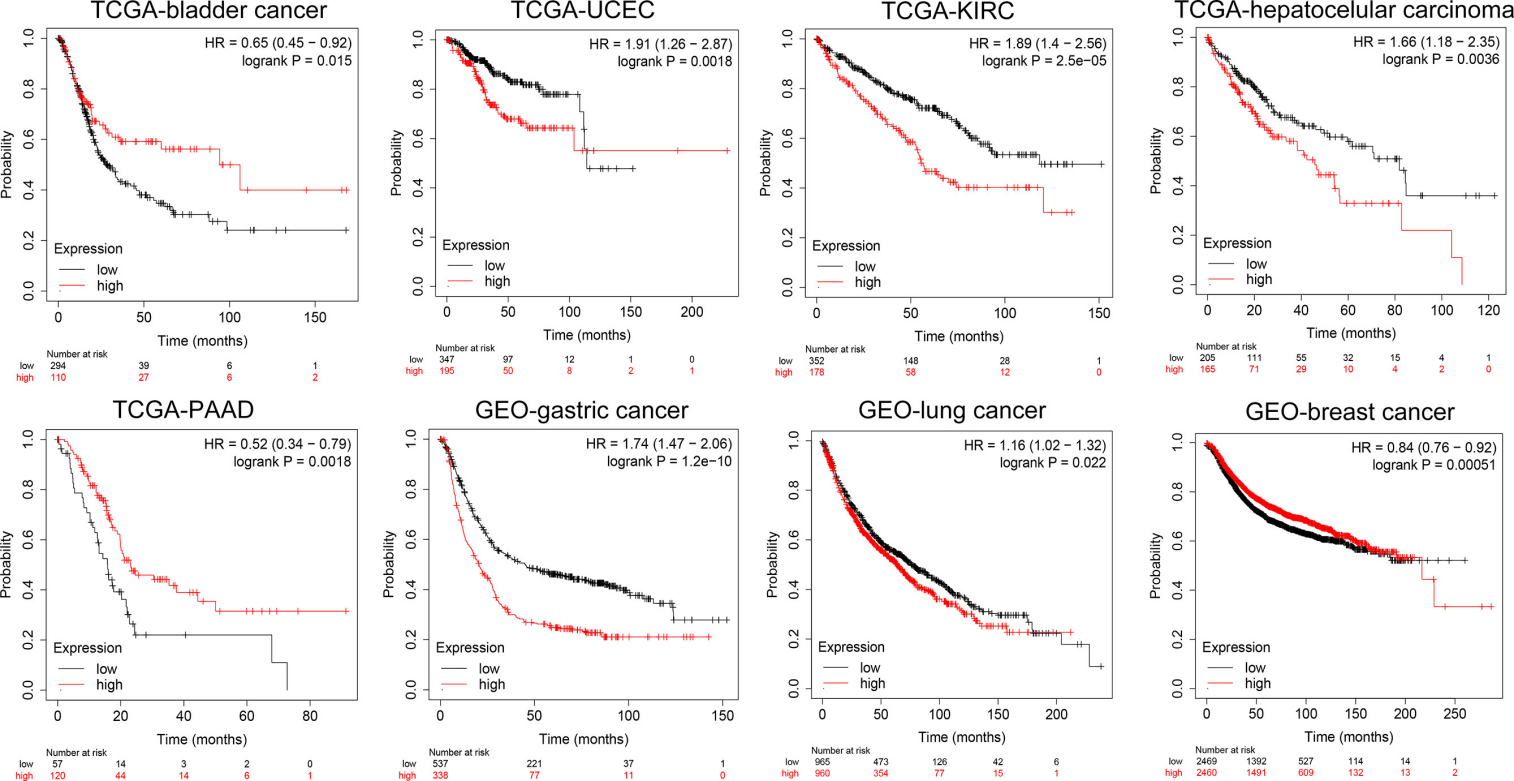
Subgroup survival analysis indicated high expression of KCC2 is associated with poor survival for pan-cancer patients. Bladder cancer (HR = 0.65, *p* = 0.015, TCGA), PAAD (HR = 0.52, *p* = 0.0018, TCGA), breast cancer (HR = 0.84, *p* = 0.00051, *n* = 4,929, GEO), UCEC (HR = 1.91, *p* = 0.0018, TCGA), KIRC (HR = 1.89, *p* < 0.001, TCGA), LIHC (HR = 1.66, *p* = 0.0036, TCGA), gastric cancer (HR = 1.74, *p* < 0.001, *n* = 875, GEO), and lung cancer (HR = 1.16, *p* = 0.022, *n* = 1,925, GEO).

Furthermore, we conducted a detailed Kaplan–Meier survival analysis of the specific effects of KCC2 (SLC12A5) and NKCC1 (SLC12A2) on patient survival in various cancers. As shown in **Figure 4**, we revealed that KCC2 has an marked impact on the prognosis of a variety of cancer patients. In bladder cancer (HR = 0.65, *p* = 0.015), PAAD (HR = 0.52, *p* = 0.0018), and breast cancer (HR = 0.84, *p* = 0.00051, *n* = 4,929, GEO), the higher the expression of KCC2, the better the prognosis of the patient. However, in some tumors, KCC2 has the opposite predictive prognostic implications, such as UCEC (HR = 1.91, *p* = 0.0018), KIRC (HR = 1.89, *p* < 0.001), LIHC (HR = 1.66, *p* = 0.0036), gastric cancer (HR = 1.74, *p* < 0.001, *n* = 875, GEO), and lung cancer (HR = 1.16, *p* = 0.022, *n* = 1,925, GEO).

**Figure 5:**
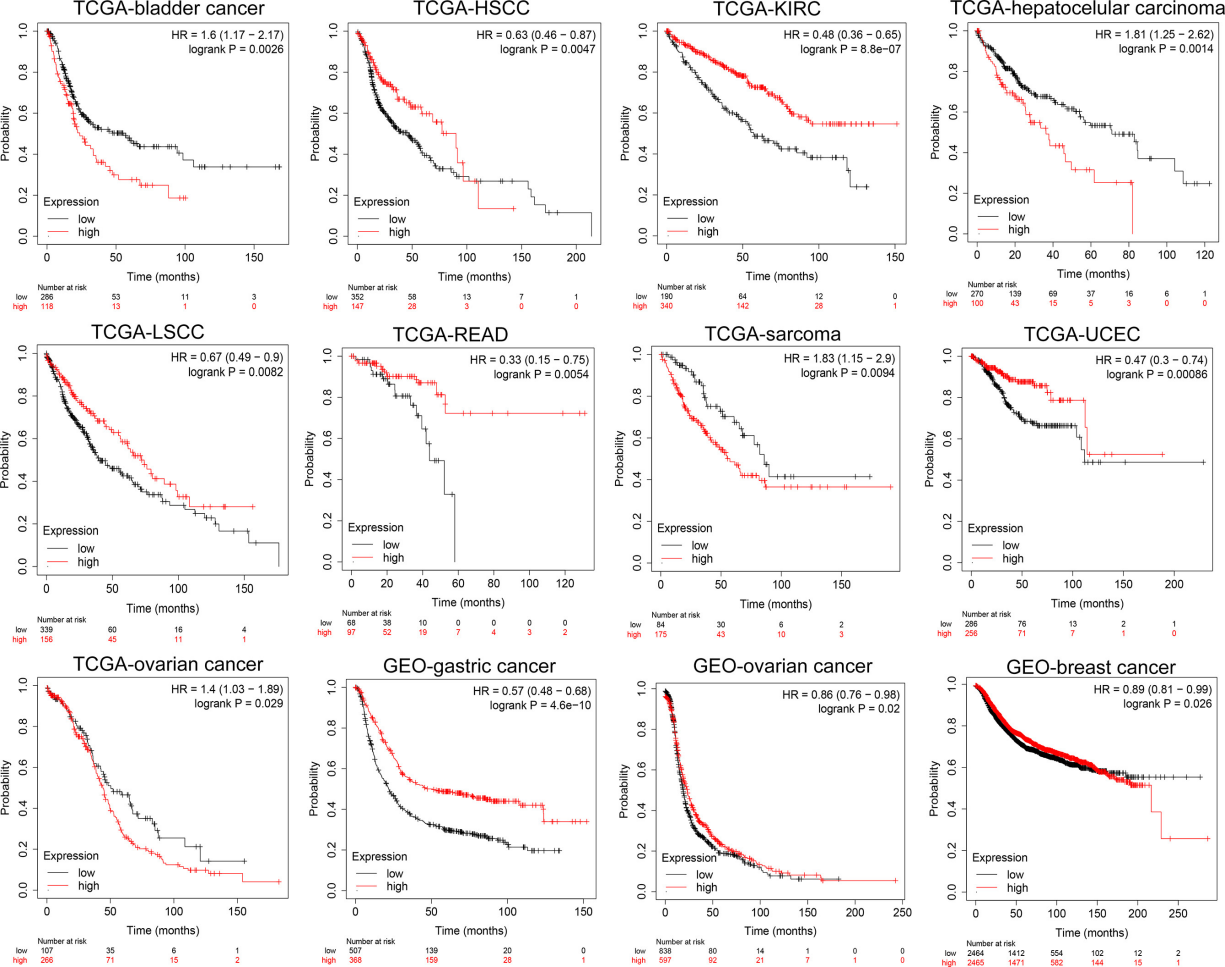
Subgroup survival analysis indicated that high expression of NKCC1 is associated with poor survival for pan-cancer patients. Bladder cancer (HR = 1.60, *p* = 0.0026, TCGA), LIHC (HR = 1.81, *p* = 0.0014, TCGA), sarcoma (HR = 1.83, *p* = 0.0094, TCGA), ovarian cancer (HR = 1.41, *p* = 0.029, TCGA), HSCC (HR = 0.61, *p* = 0.0047, TCGA), KIRC (HR = 0.48, *p* < 0.001, TCGA), LSCC (HR = 0.67, *p* = 0.0082, TCGA), READ (HR = 0.33, *p* = 0.0054, TCGA), UCEC (HR = 0.47, *p* < 0.001, TCGA), gastric cancer (HR = 057, *p* < 0.001, *n* = 875, GEO), and breast cancer (HR = 0.89, *p* = 0.026, *n* = 4,929, GEO).

At the same time, we analyzed the prognostic implications of the expression of NKCC1 in pan-cancer on patients. In **Figure 5**, we found that the higher the expression of NKCC1, the worse the prognosis of patients with bladder cancer (HR = 1.60, *p* = 0.0026), LIHC (HR = 1.81, *p* = 0.0014), sarcoma (HR = 1.83, *p* = 0.0094), and ovarian cancer (HR = 1.41, *p* = 0.029) from TCGA database. In HSCC (HR = 0.61, *p* = 0.0047), KIRC (HR = 0.48, *p* < 0.001), LSCC (HR = 0.67, *p* = 0.0082), READ (HR = 0.33, *p* = 0.0054), UCEC (HR = 0.47, *p* < 0.001), gastric cancer (HR = 057, *p* < 0.001, *n* = 875, GEO), and breast cancer (HR = 0.89, *p* = 0.026, *n* = 4,929, GEO), the higher the expression of NKCC1, the better the prognosis of the patient. Overall, in nearly 20,000 pan-cancer samples with overall survival information, KCC2 and NKCC1 showed different prognostic values in different cancer types, suggesting that CCCs have inconsistent tumorigenesis regulatory mechanisms in cancers.

### DNA Variation Landscape of KCC2 and NKCC1 and Their Prognostic Implications in Pan-Cancer

**Figure 6:**
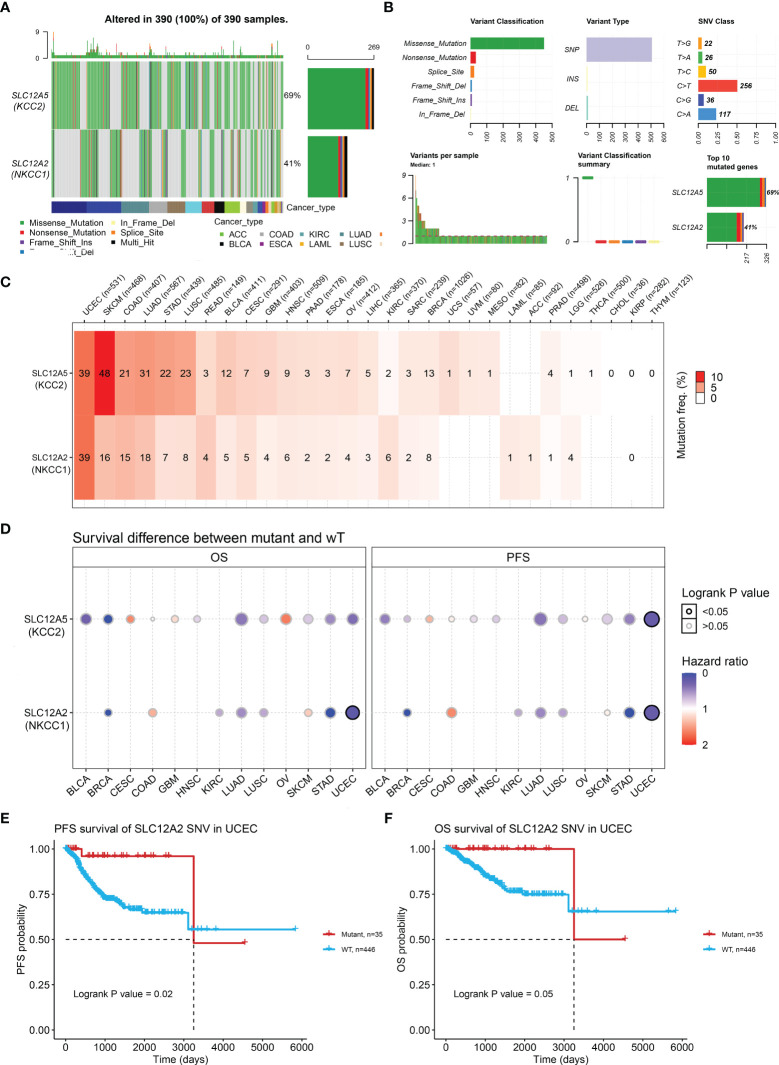
KCC2 and NKCC1 mutation analysis in pan-cancer from TCGA. **(A)** Common gene mutation frequency in pan-cancer was explored according to differential KCC2 and NKCC1 expression in 390 cases. **(B)** The variant classification, variant type, and SNV category of KCC2 and NKCC1. **(C)** The mutation frequency of KCC2 and NKCC1. UCEC (39%), SKCM (48% for KCC2, 16% for NKCC1), COAD (21% for KCC2, 15% for NKCC1), LUAD (31% for KCC2, 18% for NKCC1), and STAD (22% for KCC2, 7% for NKCC1). **(D)** The effects of wild-type and mutant types of KCC2 and NKCC1 on the prognosis of cancer patients. **(E)** The PFS survival prognostic value of NKCC1 mutations for UCEC patients (*p* = 0.02). **(F)** The OS survival prognostic value of NKCC1 mutations for UCEC patients (*p* = 0.05).

Next, we examined the DNA variation profiles of CCCs in pan-cancer. As shown in **Figure 6A**, we found 390 cases of mutations of KCC2 (SLC12A5) and NKCC1 (SLC12A2) in cancers. **Figure 6B** summarizes the variant classification, variant type, and SNV category of KCC2 and NKCC1. Then, we explored the mutation frequency of KCC2 and NKCC1 mutations in each tumor based on TCGA (**Figure 6C**). The results showed that the DNA alteration frequency of KCC2 and NKCC1 reached the highest in UCEC (39%), followed by SKCM (48% for KCC2, 16% for NKCC1), COAD (21% for KCC2, 15% for NKCC1), LUAD (31% for KCC2, 18% for NKCC1) and STAD (22% for KCC2, 7% for NKCC1). Furthermore, we analyzed the effects of wild-type and mutant types of CCCs on the prognosis of cancer patients (**Figure 6D**). Interestingly, we found that the transition of wild-type and mutant types of NKCC1 had the greatest impact on the survival of UCEC patients. Next, we explored the specific prognostic value of NKCC1 mutations for UCEC patients (**Figures 6E, F**). The results demonstrated that UCEC patients with NKCC1^MUT^ were under significantly better PFS (*p* = 0.02) and OS (*p* = 0.05).

In order to further explore the effect of DNA alteration on tumorigenesis, we verified the expression of correlation of CNV with transcriptional expression of KCC2 and NKCC1 (**Supplementary Figure 1A**). Cancers, such as BRCA, UCEC, and STAD, showed significantly consistent correlation (*p* < 0.05). As shown in **Supplementary Figure 1B**, we found that the CNV and expression of KCC2 had a significantly positive relationship in ACC, LUSC, and STAD (*p* < 0.001), while the CNV and expression of NKCC1 had a markedly positive relationship in OV, BRCA, and UCEC (*p* < 0.001).

### Landscape and Prognostic Implications of CNV of CCCs in Pan-Cancer

**Figure 7:**
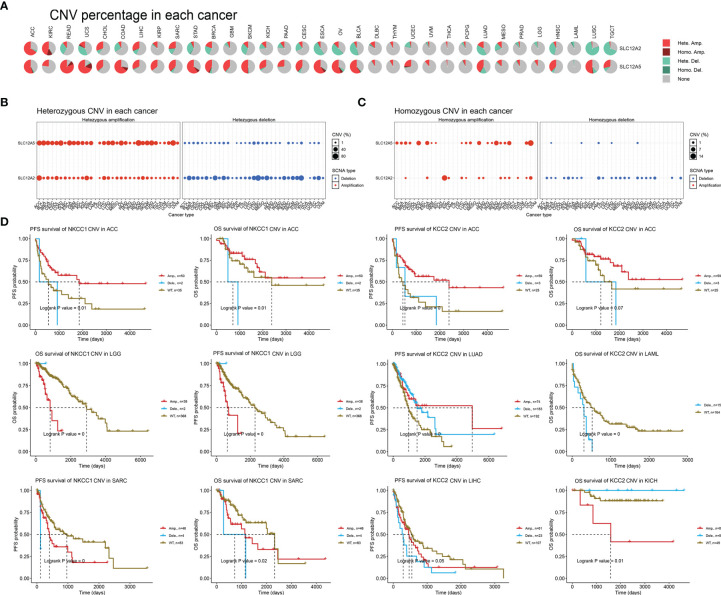
The landscape and prognostic significance of CNV of KCC2 and NKCC1 in pan-cancer from TCGA. **(A)** Association between KCC2 and NKCC1 heterozygotes and heme amplification and deletion. **(B)** CNV characteristics of KCC2 in pan-cancer patients (heterozygous or heme amplification or deletion). **(C)** CNV characteristics of NKCC1 in pan-cancer patients (heterozygous or heme amplification or deletion). **(D)** KCC2 and NKCC1 with high frequency of CNV in pan-cancer and the impact on the prognosis of cancer patients.

After determining the DNA variation profiles of CCCs in cancers, we determined whether CNV of CCCs has the prominent clinical characteristics in pan-cancer. First, we explored the heterozygous and homozygous amplification and deletion of KCC2 (SLC12A5) and NKCC1 (SLC12A2) (**Figure 7A**). It showed the CNV characteristics (heterozygous or homozygous modifications or deletion) of KCC2 and NKCC1 in pan-cancer patients. The CNV of KCC2 is mainly reflected in Hete.Amp and Hete.Del, and the CNV of NKCC1 is mainly reflected in Hete.Amp (**Figures 7B, C**). In pan-cancer patients, the proportion of heterozygous CNV for KCC2 and NKCC1 is extraordinarily high, and KCC2 is mainly a form of heterozygous amplification, while NKCC1 is mainly a form of heterozygous deletion.

Next, we found that KCC2 (SLC12A5) and NKCC1 (SLC12A2) have a high frequency of CNV in pan-cancer, and significantly interfere with prognosis for cancer patients (**Figure 7D**). For example, in ACC, the amplification of NKCC1 significantly led to better patient survival. The survival of the NKCC1^WT^ group and the CNV deletion group was significantly worse than that of the amplification group, and the OS and PFS of ACC patients were significantly worse. For LGG patients, the OS of the amplification group of NKCC1 was significantly worse than that of the NKCC1^WT^ group. For SARC patients, the CNV of NKCC1 will significantly decrease the OS and PFS of the patients. Compared with the CNV-altered group, patients in the KCC2^WT^ group mainly suffered a significantly worse prognosis. Interestingly, the CNV deletion of KCC2 in LAML and LIHC markedly predict worse prognosis for cancer patients, while the amplification of CNV could promote significantly worse prognosis of KICH patients. Overall, we found a close relationship between the CNV and transcriptional profiles of KCC2 and NKCC1 and emphasized their clinical implications for pan-cancer patients.

### Mutation Landscape of KCC2 and NKCC1 in 10,967 Pan-Cancer From TCGA Database

**Figure 8:**
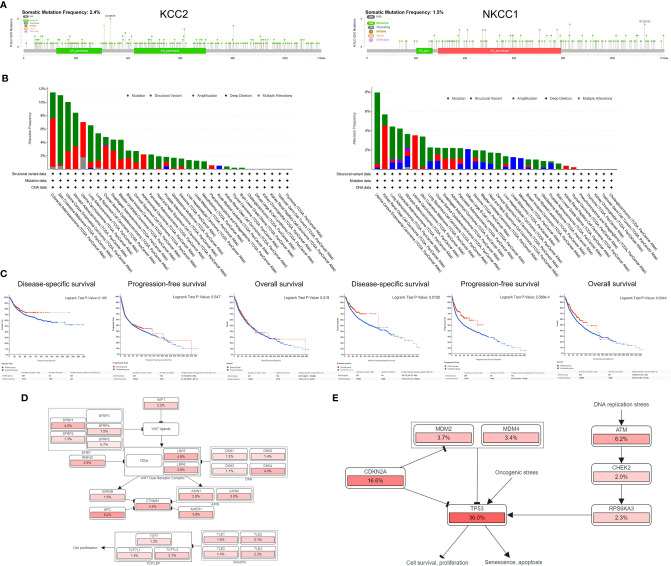
Analysis of mutation maps of KCC2 and NKCC1 among 10,967 pan-cancer patients. **(A)** Analysis of somatic mutations in KCC2 and NKCC1 and their main mutation sites (somatic mutations rate of KCC: 2.4%; somatic mutations rate of NKCC1: 1.5%). **(B)** Analysis of the change frequency of KCC2 and NKCC1 in pan-cancer. **(C)** The difference in survival between the KCC2 and NKCC1 changed and unchanged groups in pan-cancer patients, including DSS (*p* = 0.019), PFS (*p* < 0.001), and OS (*p* = 0.034). **(D)** Major functional contributors related to KCC2 and NKCC1 mutations.

After discovering that DNA variation of KCC2 (SLC12A5) and NKCC1 (SLC12A2) affect the prognosis of cancer patients, we conducted a specific analysis of CCC mutation in cancer samples from TCGA database. In **Figure 8A**, we found that the somatic mutation frequency of KCC2 mutation was 2.4% and NKCC1 mutation was 1.5% among 10,967 pan-cancer from TCGA database. The main mutation site of KCC2^MUT^ is in G332E/R, mainly in the AA_permease domain protein; the main mutation site of NKCC1^MUT^ is in R1133/Q, mainly in the AA_permease domain protein. Next, we investigate mutation, structure variant, amplification deletion, and multiple alteration frequency of CCCs among 10,967 pan-cancer from TCGA database. In **Figure 8B**, the results suggested that KCC2 has the highest alteration frequency in colorectal adenocarcinoma, and amplifier accounts for the majority. KCC2 has a higher alteration frequency in skin cutaneous melanoma and uterine corpus endometrial carcinoma. Besides, NKCC1 has the highest alteration frequency in uterine corpus endometrial carcinoma, and mutation accounts for the majority. NKCC1 has a higher alteration frequency in kidney renal clear cell carcinoma, lung adenocarcinoma, and colorectal adenocarcinoma. In general, the DNA alteration patterns of CCCs widely exist in pan-cancer.

Importantly, we analyzed the survival difference between the altered and unaltered group of KCC2 (SLC12A5) and NKCC1 (SLC12A2) among 10,967 pan-cancer patients (**Figure 8C**), including disease-specific survival (DSS), PFS, and OS. Although there was no statistically significant survival difference between KCC2 mutation and the wild-type (WT) group (*p* < 0.05), we found that NKCC1^MUT^ could prominently prolong DSS (*p* = 0.019), PFS (*p* = 2.59e-04), and OS (*p* = 0.034) compared with NKCC1^WT^ cancer patients. Then, we comprehensively studied the main functional contributors associated with the mutation of KCC2 and NKCC1, and found that KCC2^MUT^ and NKCC1^MUT^ were significantly involved in multiply malignant biological processes, such as WNT ligands, SFRP, WNT dual receptor complex, DNA replication stress and cell proliferation, and apoptosis (**Figures 8D, E**).

### Mutation Landscape of KCC2 and NKCC1 in 48,834 Pan-Cancer Samples From 188 Studies

**Figure 9:**
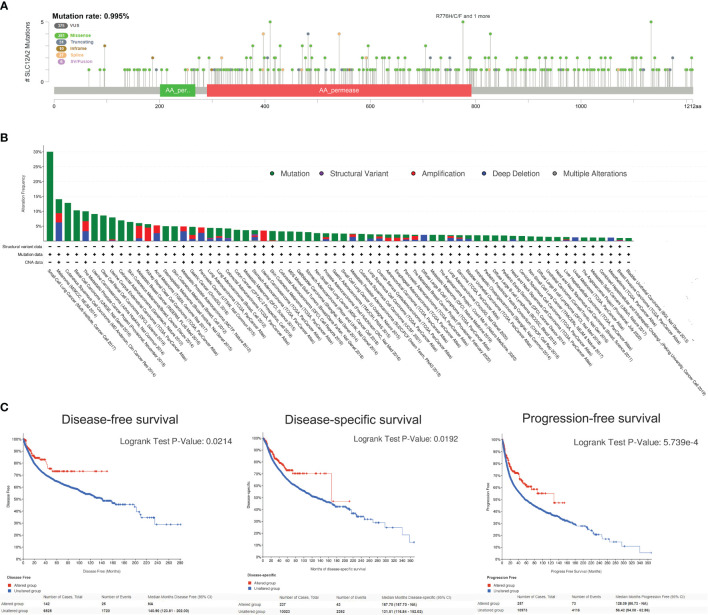
NKCC1 mutations in pan-cancer samples in 48,834 pan-cancer from 166 independent studies. **(A)** The somatic mutation frequency of NKCC1 mutation and the main mutation site in pan-cancer samples (somatic mutations rate of NKCC1: 0.995%). **(B)** Mutation frequency and DNA structure variations NKCC1 in pan-cancer. **(C)** NKCC1^MUT^ could prominently prolong DFS (*p* = 0.021), DSS (*p* = 0.019), and OS (*p* = 5.74e-04) compared with NKCC1^WT^ cancer patients. NA, Not Available.

Since the NKCC1 mutation has a prominently prognostic value on pan-cancer patients, we further analyzed the KCC2 (SLC12A5) and NKCC1 (SLC12A2) mutation, and we then queried 48,834 samples in 188 studies for a comprehensive analysis. As shown in **Figure 9A** and **Supplementary Figure 2A**, we found that the somatic mutation frequency of NKCC1 mutation was 0.995% and KCC2 mutation was 1.96% among 48,834 pan-cancer samples from 66 databases. We found that the main mutation site of NKCC1 is R776H/C/F mainly located in the AA_permease domain protein; the main mutation site of KCC2 is G332E/R. In **Figure 9B**, we found that NKCC1 has the highest frequency of changes in small-cell lung carcinoma, with amplifiers accounting for the majority. NKCC1 has a higher frequency of changes in small-cell lung cancer, melanoma, and cutaneous cell carcinoma. KCC2 has the highest frequency of changes in metastatic melanoma, with amplifiers accounting for the majority in gastric cancer. KCC2 has a higher frequency of mutations in skin cancer, such as basal cell carcinoma and cutaneous squamous cell carcinoma (**Supplementary Figure 2B**).

Importantly, we analyzed the survival difference between the altered and unaltered group of KCC2 (SLC12A5) and NKCC1 (SLC12A2) among cancer patients from 66 databases (**Figure 9C** and **Supplementary Figure 2C**). KCC2 mutation significantly predicted better disease-free survival (DFS) compared with KCC2^WT^ cancer patients (*p* = 0.0122). Although there were no statistically significant PFS and OS difference between KCC2 mutation and the WT group (*p* < 0.05), we found that NKCC1^MUT^ could prominently prolong DFS (*p* = 0.021), DSS (*p* = 0.019), and OS (*p* = 5.74e-04) compared with NKCC1^WT^ cancer patients.

### Correlation Analysis of KCC2 and NKCC1 With Immune Cell Infiltration in TME

**Figure 10:**
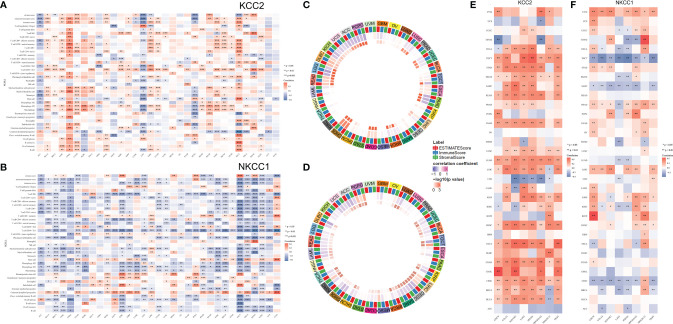
Correlation analysis of KCC2 and NKCC1 with immune cells infiltration in TME of cancers. **(A)** Enrichment scores of all TME elements and KCC2 and NKCC1 in pan-cancer. **(B)** The relationship between the expression of NKCC1 and the number of immune cells. **(C)** The circus plot showed the correlation between expression of KCC2 and the estimated score, matrix score, and immune score in pan-cancer. **(D)** The circus plot showed the correlation between expression of NKCC1 and the estimated score, matrix score, and immune score in pan-cancer. **(E)** The relationship between KCC2 expression and immune checkpoint genes in pan-cancer. **(F)** The relationship between NKCC1 expression and immune checkpoint genes in pan-cancer.

Next, we analyzed all TIME elements and the enrichment scores of KCC2 (SLC12A5) and NKCC1 (SLC12A2) in pan-cancer, and found that the expression of KCC2 was significantly correlated with the increase in the number of immune cells, including B cells, Th2 cells, and M1 macrophages (**Figure 10A**). The expression of NKCC1 was significantly correlated with the decrease in the number of immune cells, including CD8^+^ T cell, NK cell, and CD4^+^ T cell (**Figure 10B**). Next, we scored and compared the Estimate, Stromal, and Immune score of KCC2 and NKCC1 in pan-cancer (**Figures 10C, D**).

In addition, we studied the relationship between the expression of KCC2 (SLC12A5) and NKCC1 (SLC12A2) in pan-cancer and immune checkpoint genes, including SIGLEC15, IDO1, CD274, HAVCR2, PDCD1, CTLA4, LAG3, and PDCD1LG2. Interestingly, both KCC2 and NKCC1 are significantly related to tumor-infiltrating immune cells, including tumor purity and immune checkpoint molecules such as PD-L1, CTLA-4, LAG-3, and TIGIT in ccRCC. Overall, KCC2 and NKCC1 expression positively correlated with the immune checkpoint molecules (**Figures 10E, F**).

### Differential KCC2 and NKCC1 Expression From Multiply Cohorts

**Figure 11:**
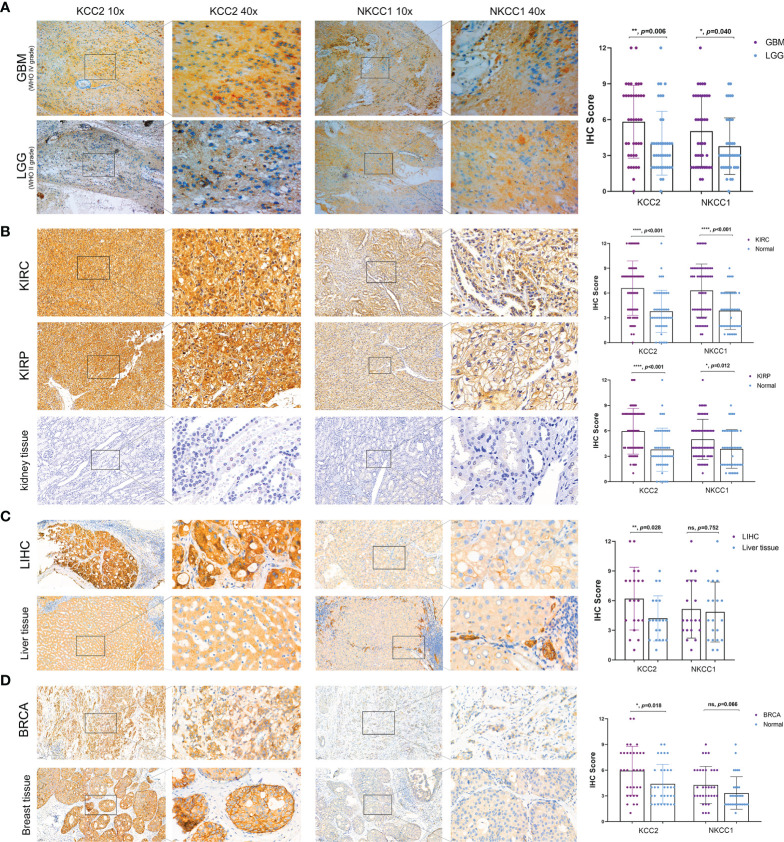
Significantly altered protein expression of KCC2 and NKCC1 based on three real-world cohorts (AHYMUN, FUSCC, and PHZU cohorts). **(A)** KCC2 and NKCC1 expression was significantly elevated in 40 GBM (WHO IV Grade) compared with 40 LGG (WHO II Grade) samples from the AHYMUN (*p* < 0.05). **(B)** In 70 clear cell renal cell carcinoma (KIRC), 50 papillary cell renal cell carcinoma (KIRP), and 50 normal kidney tissues from FUSCC cohort, KCC2 and NKCC1 expression were significantly elevated in kidney tumors compared with normal tissues (*p* < 0.05). **(C)** We compared 20 paired liver cancer and normal samples from PHZU cohort, and found significantly elevated KCC2 expression (*p* = 0.028) and insignificant expression difference of NKCC1 expression (*p* = 0.752) in liver cancer samples “ns” represents “not significant”. **(D)** The expression of KCC2 was significantly higher in 33 breast cancer tissue than in normal tissues (*p* = 0.018). *p < 0.05, **p < 0.01, ***p < 0.001.

In order to verify our hypothesis and predictive results of bioinformatics, we performed IHC analysis of KCC2 (SLC12A5) and NKCC1 (SLC12A2) to reveal the staining distribution in tumor and adjacent normal tissues of glioma (from AHYMUN cohort), renal cell carcinoma (from FUSCC cohort), and liver and breast cancer (from PHZU cohort). The scatter plot of the IHC scores revealed that KCC2 and NKCC1 expression was significantly elevated in 40 GBM (WHO IV Grade) compared with 40 LGG (WHO II Grade) samples from the AHYMUN (*p* < 0.05; **Figure 11A**), suggesting that the higher the expression of CCCs, the more aggressive the malignancy of patients with glioma. Besides, we examined 70 clear cell renal cell carcinoma (KIRC), 50 papillary cell renal cell carcinoma (KIRP), and 50 normal kidney tissues from the FUSCC cohort, and compared the expression of KCC2 and NKCC1 in tumor and normal kidney samples. We found that the expression of KCC2 and NKCC1 was significantly elevated in kidney tumors compared with normal tissues (*p* < 0.05; **Figure 11B**). Next, we compared 20 paired liver cancer and normal samples from PHZU cohort, and found significantly elevated KCC2 expression (*p* = 0.028) and insignificant expression difference of NKCC1 expression (*p* = 0.752) in liver cancer samples (**Figure 11C**). Similarly, in breast cancer (**Figure 11D**), we also found that the expression of KCC2 was significantly higher in 33 breast cancer tissue than in normal tissues (*p* = 0.018). Overall, through IHC validation analysis of KCC2/NKCC1 in tumor and normal tissues from multiple cohorts, we found the significantly differential transcriptional expression pattern of cation-chloride cotransporters in pan-cancer and normal tissues, suggesting the potential involvement of channels in the occurrence and progression of cancers.

### Prognostic Validation of KCC2 and NKCC1 Expression in 232 KIRC Patients From a Real-World Cohort

**Figure 12:**
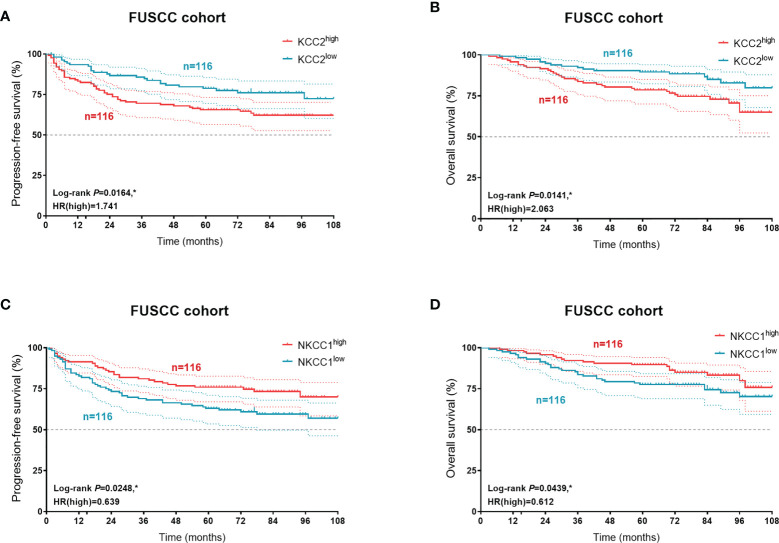
Prognostic validation of KCC2 and NKCC1 expression in 232 KIRC patients from a real-world cohort. In order to verify our hypothesis and predictive implications of KCC2 **(A, B)** and NKCC1 **(C, D)** expression, we enrolled 232 patients with follow-up and protein expression data using Kaplan–Meier methods. *p < 0.05.

In order to verify our hypothesis and predictive implications of KCC2 (SLC12A5) and NKCC1 (SLC12A2) expression, we enrolled 232 patients with follow-up and protein expression data from the FUSCC cohort (**Figure 12**). It suggested that elevated KCC2 expression significantly correlated with poor PFS (*p* = 0.0164, HR = 1.741) and OS (*p* = 0.0141, HR = 2.063), which is inconsistent with the prognostic value of KCC2 in the public database. Besides, we found that higher NKCC1 expression prominently predicts shorter PFS (*p* = 0.0248, HR = 0.639) and OS (*p* = 0.0439, HR = 0.612) for 232 patients with KIRC.

## Discussion

CCCs can catalyze K^+^, Na^+^, and Cl^-^ to carry out electrically neutral transport in various ratios (30, 31). CCCs play a key role in regulating the osmotic pressure and water balance of cells and organs (32, 33). NKCC1 is Na^+^-dependent and transports K^+^, Na^+^, and Cl^-^ in a ratio of 1:2. KCC2 is Na^+^-independent and transports K^+^ and Cl^-^ unidirectionally in a ratio of 1:1 (5, 34). In our study, we found that SLC12A2 (KCC2) and SLC12A5 (NKCC1) were highly expressed in most tumors by data mining in a database with a sufficiently large sample size. In many tumors, the difference in the expression of SLC12A2 (KCC2) and SLC12A5 (NKCC1) was statistically significant (*p* < 0.001).

Research and analysis on the role of ion transporters and channels in cancer have only begun in the past 20 years (8, 10, 35). Some studies have found that ion channels play a role in certain cancers. For example, activating K^+^ channels can accelerate the progression of breast cancer in G1 stage (13); Ca^2+^ channels can significantly affect the cell death of insulinoma cells (36); Na^+^ voltage-gated channels are overexpressed in highly malignant breast and prostate cancer cells (37).

NKCC is clinically used as a loop diuretic of sulfonamides, such as furosemide and bumetanide (38). We suspect that this is the reason why NKCC1 is significantly expressed in urinary tract tumors such as KIRC and KIRP. Studies have also found that NKCC1 is related to interneurons in the brain (39, 40) and plays a certain role in controlling epilepsy (38, 41). The regulatory role of solute carrier family 12 members in tumor progression and immune responses has been rarely reported, and recent studies have found that SLC12A8 plays a critical role in bladder cancer progression and EMT (42). Some new studies believe that NKCC1 is an essential ion cotransporter in the progression of glioma (43). The transcription mechanism of KCC2 gene expression regulation is mostly unclear. Studies have found that KCC2 plays a role in promoting malignant cervical cancer cells (44). In our research, it was also found that the expression level of KCC2 in several epithelial cancers (breast cancer, cervical cancer, colorectal cancer, kidney cancer, lung cancer, ovarian cancer, pancreatic cancer, and prostate cancer) was significant. This means that KCC2 may be a biomarker for epithelial cancer. In conclusion, our research found that KCC2 plays a very important role in tumor immune regulation and regulation of cell migration. The 1.5% mutation frequency of KCC2 and NKCC1 in all samples in the pan-cancer study is a low percentage, so we will conduct further analysis by studying multicenter samples in the next study.

Ion transporters are important in regulating ion homeostasis, cell volume, and cell signal transduction under physiological conditions. Through our research, we can find that KCC2 and NKCC1, the two key ion transport genes, have become important participants in the invasion and development of various cancers. Therefore, KCC2 and NKCC1 have great potential as new targets for the treatment of multiple malignant tumors including LGG, GBM, KIRC, KIRP, and BRCA.

## Conclusion

This study first investigated the molecular and clinical role of cation-chloride cotransporters, and illustrated the significant connection among KCC2/NKCC1 expression, DNA variation profiles, prognosis, and immune-rejection tumor microenvironment of pan-cancer. Taken together, these findings indicated that KCC2/NKCC1, acting as a biomarker, could guide the molecular diagnosis and prompt novel targeted therapeutic strategies for pan-cancer patients.

## Data Availability Statement

The raw data supporting the conclusions of this article will be made available by the authors, without undue reservation.

## Ethics Statement

The studies involving human participants were reviewed and approved by People’s Hospital of Zhengzhou University, Affiliated Hospital of Youjiang Medical University for Nationalities and Fudan University Shanghai Cancer Center. The patients/participants provided their written informed consent to participate in this study.

## Author Contributions

Conceptualization: WX, WZ, XT, and WL. Data curation and formal analysis: WX, WZ, XT, AA, YW, WL, and JS. Funding acquisition: SW, WX, YQ, HZ, and DY. Investigation and methodology: WX, WZ, AA, WZ, YW, and WL. Resources and software: WL, WS, YQ, HZ, YW, and DY. Supervision: YQ, SW, HZ, and DY. Validation and visualization: WX, WL, WZ, XT, and AA. Original draft: WX, WS, YW, and WL. Editing: WS, YQ, HZ, and DY.

## Funding

This work is partially supported by Grants from the Natural Science Foundation of Henan Province (No. 222300420363), the Henan Province Medical Science and Technology Research Program (No. SB201902029), National Key Research and Development Program of China (No. 2019YFC1316000), and the Natural Science Foundation of Shanghai (No. 20ZR1413100).

## Conflict of Interest

The authors declare that the research was conducted in the absence of any commercial or financial relationships that could be construed as a potential conflict of interest.

## Publisher’s Note

All claims expressed in this article are solely those of the authors and do not necessarily represent those of their affiliated organizations, or those of the publisher, the editors and the reviewers. Any product that may be evaluated in this article, or claim that may be made by its manufacturer, is not guaranteed or endorsed by the publisher.

## Supplementary Material

Supplementary Figure 1 The correlation between copy number variation and expression of KCC2 and NKCC1 in cancers. **(A)** Correlation between CNV expression and transcriptional expression of KCC2 and NKCC1. **(B)** The effect of CNV expression and KCC2 and NKCC1 on the survival of pan-cancer patients.

Supplementary Figure 2 KCC2 mutations in 48,834 pan-cancer from 166 independent studies. **(A)** In pan-cancer, the somatic mutation frequency of KCC2 mutation and the main mutation site (somatic mutation frequency:1.96%). **(B)** Frequency of change of KCC2 in pan-cancer. **(C)** Survival difference between the KCC2^MUT^ and KCC2^WT^ group in pan-cancer using K-M methods.
