# Supplementary figures and images for: Comprehensive Analysis of the Oncogenic, Genomic Alteration, and Immunological Landscape of Cation-Chloride Cotransporters in Pan-Cancer
Source: Front Oncol. 2022 Mar 17;12:819688. doi: 10.3389/fonc.2022.819688 (PMC8968682; doi:10.3389/fonc.2022.819688)

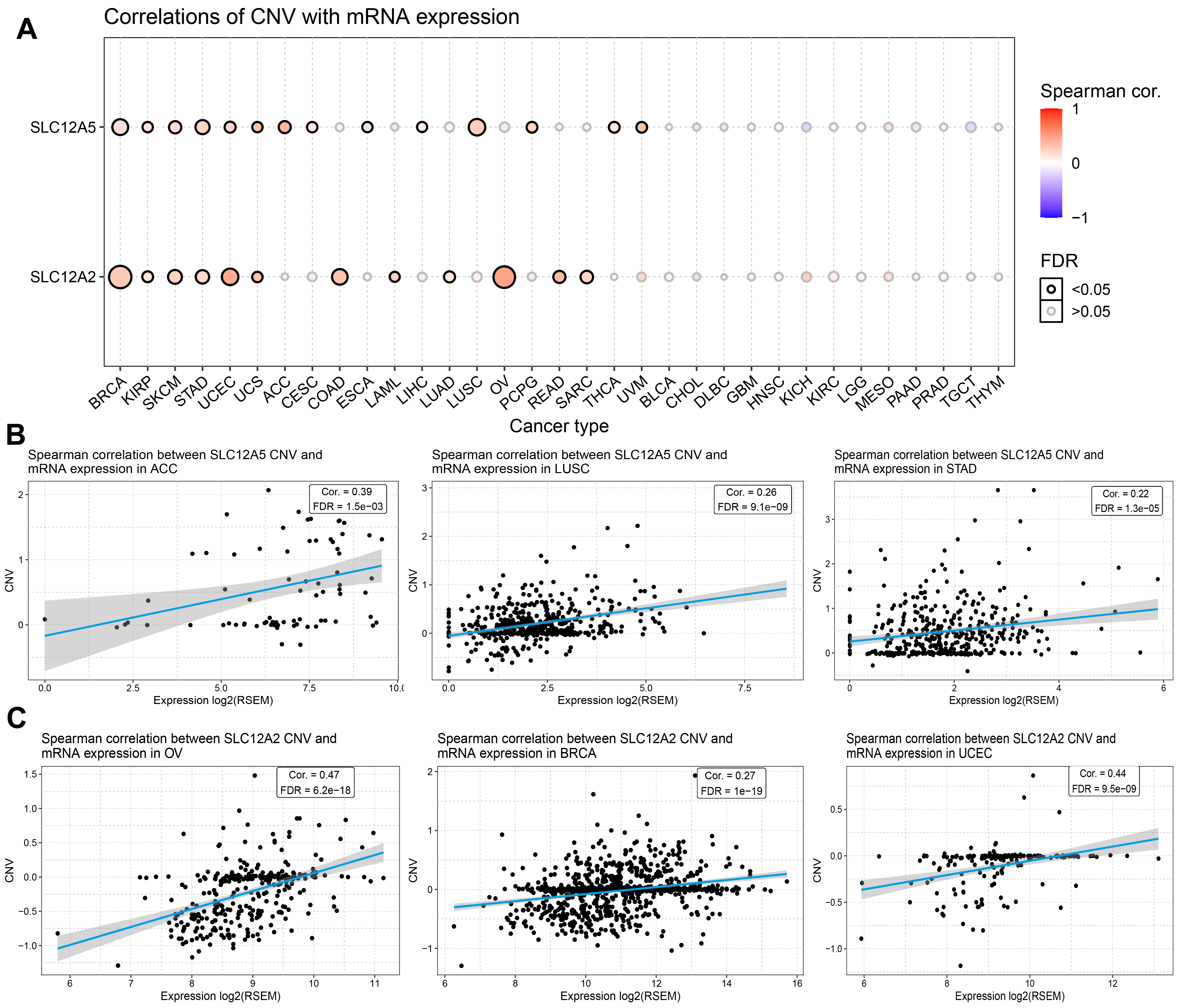

Supplement: Supplementary Figure 1 — The correlation between copy number variation and expression of KCC2 and NKCC1 in cancers. (A) Correlation between CNV expression and transcriptional expression of KCC2 and NKCC1. (B) The effect of CNV expression and KCC2 and NKCC1 on the survival of pan-cancer patients. [file Image_1.tif]

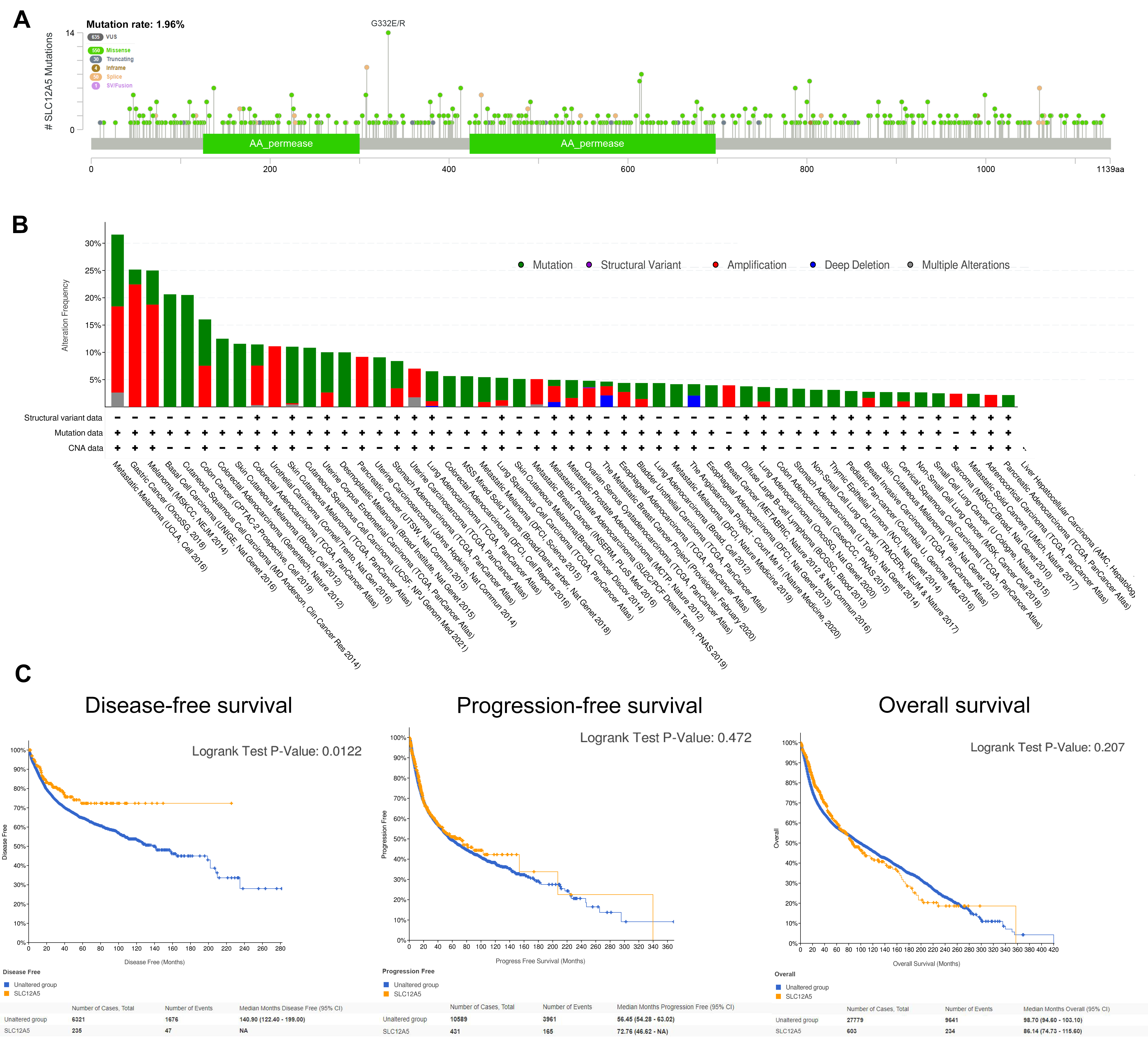

Supplement: Supplementary Figure 2 — KCC2 mutations in 48,834 pan-cancer from 166 independent studies. (A) In pan-cancer, the somatic mutation frequency of KCC2 mutation and the main mutation site (somatic mutation frequency:1.96%). (B) Frequency of change of KCC2 in pan-cancer. (C) Survival difference between the KCC2MUT and KCC2WT group in pan-cancer using K-M methods. [file Image_2.tif]
